# Potential Role of S-Palmitoylation in Cancer Stem Cells of Lung Adenocarcinoma

**DOI:** 10.3389/fcell.2021.734897

**Published:** 2021-09-21

**Authors:** Yitong Zhang, Fenglan Li, Kexin Fu, Xiqing Liu, I-Chia Lien, Hui Li

**Affiliations:** ^1^Department of Biochemistry and Molecular Biology, Harbin Medical University, Harbin, China; ^2^Department of Gastrointestinal, The First Hospital of Jilin University, Changchun, China; ^3^The State Key Laboratory of Networking and Switching Technology, Beijing University of Posts and Telecommunications, Beijing, China; ^4^Insight Genomics Inc., National Cheng Kung University, Tainan, Taiwan

**Keywords:** ZDHHC5, INCENP, S-palmitoylation, mRNAsi, cancer stem cell, lung adenocarcinoma, machine learning

## Abstract

S-palmitoylation, catalyzed by a family of 23 zinc finger Asp-His-His-Cys (DHHC) domain-containing (ZDHHC) protein acyltransferases localized on the cell membrane. However, stemness genes modulated by ZDHHCs in lung adenocarcinoma (LUAD) remain to be defined. Previously, we have constructed a network of cancer stem cell genes, including INCENP, based on mRNA stemness indices (mRNAsi) of LUAD. INCENP has the function of a chromosomal passenger complex locating to centromeres, which is performed by the conserved region of its N-terminal domain. INCENP protein with a deletion of the first non-conserved 26 amino acid sequence failed to target centromeres. However, the exact function of the deleted sequence has not been elucidated. To identify novel cancer stem cell-relevant palmitoylated proteins and responsible ZDHHC enzymes in LUAD, we analyzed multi-omics data obtained from the database of The Cancer Genome Atlas (TCGA), Gene Expression Omnibus (GEO), Clinical Proteomic Tumor Analysis Consortium (CPTAC), and the Human Protein Atlas (HPA). ZDHHC5 is distinguished from the ZDHHC family for being up-regulated in mRNA and protein levels and associated with malignant prognosis. ZDHHC5 was positively associated with INCENP, and the correlation score increased with LUAD stages. CSS-Palm results showed Cys^15^ was the S-palmitoylation site of INCENP. Interestingly, Cys^15^ locates in the 1–26 aa sequence of INCENP, and is a conserved site across species. As INCENP is a nuclear protein, we predicted that the nuclear localization signal of ZDHHC5 was specific to the importin αβ pathway, and the result of immunofluorescence proves that ZDHHC5 is located in the nucleoplasm, in addition to the plasma membrane. Therefore, our study indicates the S-palmitoylation of INCENP mediated by ZDHHC5 as a potential mechanism of S-palmitoylation to modulate CSCs in LUAD.

## Introduction

Given the increasing changes in attitude toward the risks of exposure to cigarette smoking and increasing air pollution, the prevalence of lung squamous cell carcinoma cases has declined; lung adenocarcinoma (LUAD) became the most common lung cancer in 1998–2002 (Lortet-Tieulent et al., 2014; Travis et al., 2015). Cancer incidence and mortality can be reduced by population screening, but LUAD is often diagnosed at advanced stages with disseminated metastatic tumors. Advanced LUAD are inoperable and highly resistant tumors to chemotherapy and irradiation. Despite the emerging improvements in chemoradiotherapy, targeted therapy, and immunotherapy, the 5-year survival rates remain low (7–20%), and recurrence rates remain high at 30–50% (Miyata et al., 2015). With limited effective therapeutic options in LUAD, identification of novel targets for therapy is sorely needed.

Cancer stemness properties, including self-renewal and differentiation, was initially attributed to normal stem cells (Malta et al., 2018). Cancer stem cells (CSCs) are responsible for cancer treatment resistance, leading to relapse, disease progression, and eventually systemic disease (Leão et al., 2017). Recent advances in high-throughput technology and machine learning have improve novel understanding of tumor heterogeneity and developed transcriptional classifications of LUAD, including molecular subtypes (Ruiz-Cordero and Devine, 2020; Wadowska et al., 2020) and immune classification (Thorsson et al., 2018). Malta et al. (2018) used an innovative one-class logistic regression machine-learning algorithm (OCLR) to analyze the molecular profiles of normal stem cell types, and they applied the OCLR-based signatures to The Cancer Genome Atlas (TCGA) datasets to obtain mRNA stemness indices (mRNAsi). Each patient of TCGA has a score of stemness index, which ranges from low (zero) to high (one) stemness. Previously, we characterized the expression of LUAD stem cell genes by mRNAsi and weighted gene co-expression network analysis (WGCNA) was used to construct a LUAD stem cell gene network (Zhang et al., 2020).

Protein lipidation events are one of an essential and diverse class of post-translational modification. S-palmitoylation is the most studied form of protein lipidations, which could reversibly attach palmitate (16-carbon) to specific cysteine residues in protein substrates. The so-called “palmitoylome” comprises palmitoylated proteins encoded by approximately 10% of the genome (Spinelli et al., 2018). Aberrant palmitoylation’s dynamic circulation might affect protein localization, accumulation, secretion, stability, and function by changing membrane affinity (Dunphy and Linder, 1998; Percherancier et al., 2001; Lanyon-Hogg et al., 2017; Jiang et al., 2018). Conventionally, palmitoylation sites were mapped by mutagenesis of candidate cysteine residues. Proteomic methods of high-throughput and tandem mass spectrometry (MS) were also applied to identifying palmitoylation sites. However, these results remained to be dissected.

In mammalian cells, the S-palmitoylation to internal cysteine residues is modulated by a family of 23 zinc finger Asp-His-His-Cys (DHHC) domain-containing (ZDHHC) protein acyltransferase (PATs) (Fukata et al., 2006). To identify novel S-palmitoylation substrates in LUAD, systemically evaluation of the ZDHHC family for the key ZDHHC is necessary. Previously, most ZDHHC proteins were found to localize to the plasma membrane, endoplasmic reticulum, and the Golgi apparatus (Ko and Dixon, 2018). However, recently, nucleoproteins of transcription factors (Chan et al., 2016), chromatin remodelers (Riccio, 2010), and histone proteins (Wilson et al., 2011) have been proposed as targets for palmitoylation (Spinelli et al., 2018). Therefore, nucleus-targeting S-palmitoylation may provide insight into novel strategies for cancer therapy.

The topic of palmitoylation in normal stem cells or CSCs is an open issue. To date, the function of palmitoylation has only been reported in neural stem cells, glioma stem cells, and epidermal growth factor receptor tyrosine kinase inhibitor (EGFR-TKI) resistance non-small cell lung cancer (NSCLC) cells. Li et al. (2012) found that in normal neural stem cells, ZDHHC5 protein expression declined within minutes following growth factor withdrawal, which led to the induction of neural differentiation in culture. It has been reported that ZDHHC5, ZDHHC17, ZDHHC18, and ZDHHC23 contribute to glioblastoma multiforme development and malignant progression by targeting glioma stem cells (GSC) self-renewal (Chen et al., 2017, 2019, 2020a). The EGFR pathway is closely associated with the stem-like properties in NSCLC (Codony-Servat et al., 2019). Palmitoylation of EGFR enhances its nuclear translocation, and EGFR palmitoylation is important to promote the growth of EGFR-TKI resistance NSCLC cells (Ali et al., 2018). Hence, identifying key ZDHHCs and their stemness substrates is greatly useful for the therapeutic application of LUAD.

To address this issue, we analyzed the ZDHHC family of S-acyltransferases to identify critical ZDHHCs involved in LUAD. ZDHHC5 was upregulated at both mRNA and protein levels in LUAD and was associated with an unfavorable prognosis. We found inner centromere protein (INCENP) is the key node connecting the LUAD stem gene network and ZDHHC5 association genes. Protein sequence analysis further predicted Cys^15^ as the S-palmitoylation site on INCENP protein. As INCENP is a nuclear protein, the nuclear localization of ZDHHC5 protein was predicted *in silico* and verified by immunofluorescence in the A549 cell line. Our work provides insights into the functions of S-palmitoylation on INCENP and suggests that inhibiting ZDHHC5 is a potential anticancer strategy for CSCs in LUAD.

## Results

### Identification of Lung Adenocarcinoma-Related ZDHHCs

**FIGURE 1:**
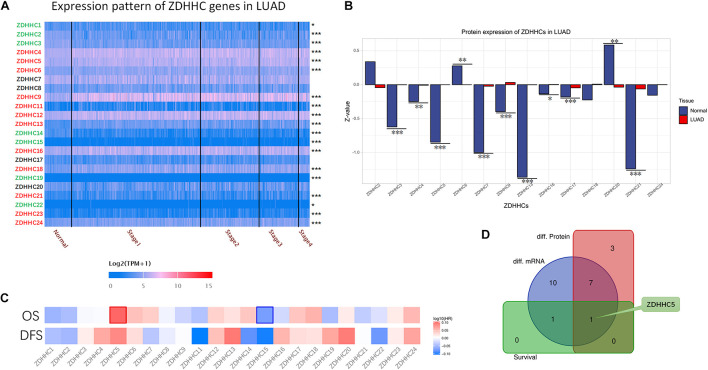
General profiling of ZDHHCs family gene expression and survival analysis in LUAD. **(A)** The heat map shows the RNA-seq results of ZDHHC mRNA in cases from the LUAD data set of TCGA. The LUAD cases are shown separately by clinical stages. The ZDHHCs coding in green indicate lower expression of the LUAD cases than normal tissues, and the red indicates higher expression. The color of scale bar presents high (red) and low (blue) expression, respectively, and the intensity of color indicates the value of mRNA expression. **(B)** The differential proteomic expression of ZDHHCs in LUAD cases from the CPTAC database. In the bar plot, the length of the bar represents the normalized expression (*Z-value*) of ZDHHC in LUAD. The color of the bar presents LUAD (red) and normal lung (blue). **(C)** Heat map of log_10_ (HR) illustrating the prognosis of ZDHHCs in LUAD patients from the TCGA database. The scale bar presents high (red) and low (blue) risk, respectively, and the intensity indicates the HR. The bounding box around the tiles represent statistically significant cancer types (HR > 1, *P* < 0.05). HR, hazard ratio; OS, overall survival; DFS, disease free survival. **(D)** The Venn diagram shows the mapping results of 3 ZDHHC gene sets. Purple, ZDHHCs that are differentially expressed at the mRNA level; red, ZDHHCs that are differentially expressed at the protein level; green, ZDHHCs with prognostic value. *P < 0.05, **P < 0.01, ***P < 0.001.

To date, 23 PATs, also known as ZDHHC proteins, expressed in mammalian cells have been discovered. Given that the function of ZDHHC in LUAD tumorigenesis is variable, we reasoned that finding suitable ZDHHC is important for studying the modulatory mechanisms of palmitoylation. To identify potential ZDHHC targets in LUAD, we analyzed the RNA-seq data from TCGA database to compare differentially expressed genes between LUAD cases and normal lung tissues. As shown in Figure 1A, 12 ZDHHCs were significantly upregulated in LUAD, and 7 ZDHHCs were significantly downregulated (fold change > 1, *P* < 0.05). We further interrogated MS-based proteomic data from the Clinical Proteomic Tumor Analysis Consortium (CPTAC) Confirmatory/Discovery cohorts to investigate ZDHHC expression at the protein level, and identified 9 upregulated and 2 downregulated (fold change > 1, *P* < 0.05) ZDHHCs (Figure 1B). Heat maps (Figure 1C) showed the hazard ratio (HR) of 23 ZDHHCs in LUAD based on overall survival (OS) and disease-free survival (DFS) results. We found that high expression of ZDHHC5 and ZDHHC15 was associated with an unfavorable prognosis and a favorable outcome, respectively. To our surprise, ZDHHC5 was the only ZDHHC with differential expression and prognostic value in LUAD (Figure 1D).

### ZDHHC5 Was Over-Expressed in Lung Adenocarcinoma and Indicated Unfavorable Prognosis

**FIGURE 2:**
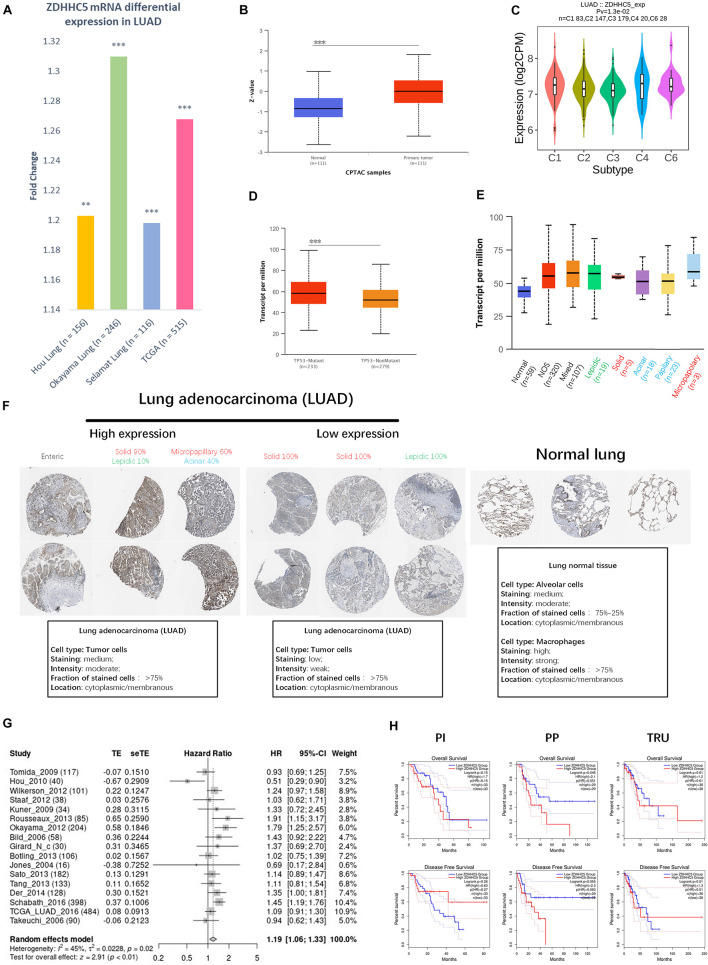
Expression of ZDHHC5 in LUAD. **(A)** Fold change of ZDHHC5 differential expression in datasets of lung adenocarcinoma from the GEO and TCGA database. **(B)** Box plot indicates the differential protein expression of ZDHHC5 in the LUAD sample from CPTAC. *Z*-value, standard deviations from the median across LUAD samples. **P < 0.01, ***P < 0.001. **(C)** Distribution of ZDHHC5 expression in LUAD across immune subtypes. **(D)** Box plot showing mRNA expression of ZDHHC5 in LUAD based on TP53 mutation status. **(E)** Box plot showing the relative transcription of ZDHHC5 in histological subtypes of LUAD patients. LUAD-not otherwise specified (NOS), LUAD mixed subtype (Mixed), lung solid pattern predominant adenocarcinoma (Solid), lung acinar adenocarcinoma (Acinar), lung micropapillary adenocarcinoma (Micropapillary), lung papillary adenocarcinoma (Papillary). **(F)** Representative immunohistochemistry results of ZDHHC5 in LUAD. We evaluated histological subtypes to address the heterogeneity of ZDHHC5 in growth patterns of LUAD and normal lung tissues. The histological subtype was classified by a clinical pathologist, and semiquantitative estimations of the different histological patterns present in 5% increments according to the 2015 WHO Classification of Lung Tumors. Enteric adenocarcinoma (Enteric); lepidic adenocarcinoma (Lepidic). **(G)** Forest plot showing adjusted analysis of ZDHHC5 survival probability on OS in LUAD patients from 17 independent studies. The summarized HR is 1.19, *P* < 0.05. CI, confidence interval. **(H)** Kaplan-Meier survival curves of OS and DFS were generated for the comparison of ZDHHC5’s prognostic value in the 3 molecular subtypes of LUAD. The threshold of significance is *P* < 0.05 in Log-rank test. PP, Proximal-proliferative; PI, proximal-inflammatory; TRU, Terminal respiratory unit.

We compared the transcriptional level of ZDHHC5 in LUAD with that in normal samples using 3 independent studies from the Gene Expression Omnibus (GEO) database. In Figure 2A, the mRNA expression of ZDHHC5 was also significantly upregulated (fold change > 1, *P* < 0.05). The mass spectrometry results of ZDHHC5 from the CPTAC database (Figure 2B) revealed the over expression of ZDHHC5 protein in LUAD. There are 5 known immune subtypes within LUAD tumors (Thorsson et al., 2018), including C1 (wound healing), C2 (IFN-γ dominant), C3 (inflammatory), C4 (lymphocyte depleted), and C6 (TGF-β dominant); overall survival analysis stratified by immune subtype indicated that C3 had the most favorable prognosis, and C6 and C4 had the poorest outcome. This immunogenomics pipeline was used to characterize these heterogeneous tumors, and we used the resulting data to explore the distribution of ZDHHC5 in immune subtypes. ZDHHC5 was slightly downregulated in C3 and was overexpressed in C4 (Figure 2C), which is in accordance with the proposed malignant role of ZDHHC5 in LUAD. We also explored the Spearman correlation of ZDHHC5 with 3 types of immune regulators and tumor-infiltrating lymphocytes in LUAD, but there was no significant association (Supplementary Figure 1A). TCGA Consortium proposed a transcriptional data-based molecular classification for LUAD, which includes 3 molecular subgroups into the clinical classification of LUAD (Cancer Genome Atlas Research Network, 2014). We performed gene expression analysis of ZDHHC5 in the 3 molecular subtypes (Supplementary Figure 1B), but there was no significant difference (*P* > 0.05). As shown in Figure 2D, the mRNA expression level of ZDHHC5 was higher in TP53-mutant LUAD cases than in the non-mutant cases.

The 2015 World Health Organization (WHO) classification of lung tumors results from an integrated multi-disciplinary approach involving radiology, molecular biology, and medical oncology (Travis et al., 2015). We analyzed the RNA-seq data of ZDHHC5 in LUAD pathological types (Figure 2E). Immunohistochemical (IHC) results of LUAD and normal lung tissue from the Human Protein Atlas (HPA) database (Uhlén et al., 2015) were obtained to be determined (Figure 2F). Basic annotation parameters were scored for the HPA samples by pathologists. ZDHHC5 was stained in over 75% of tumor cells, including medium and low expression LUADs. In lung normal tissues, ZDHHC5 was highly stained in macrophages, with a high fraction (>75%) of stained cells. However, ZDHHC5 showed medium-staining intensity in only 25–75% alveolar cells, which was lower than in the LUAD. Different pathological types represent different prognoses: lepidic represent good, acinar, and papillary standing for intermediate, and micropapillary and solid representing worse prognosis. The clinicopathological diagnosis of LUAD reports the percentage of all types in increments of 5%. Thus, we evaluated the histological subtype of the IHC results and reported the percentage of all identifiable patterns in 5% increments. This classification indicated ZDHHC5 protein expression varied within different histological subtypes, which further supported the heterogeneity of the growth patterns among LUAD. However, the IHC data was not sufficient for statistical analysis.

To confirm the significance of ZDHHC5 in the prognosis of LUAD, we performed OS analysis of ZDHHC5 in 17 independent cohorts. The survival analysis results of ZDHHC5 in these 17 study cohorts was moderately heterogeneous (*I*^2^ = 45%). As shown in Figure 2G, the HR of ZDHHC5 corrected by meta-analysis was 1.19 (*P* < 0.01), which indicated ZDHHC5 was associated with an unfavorable prognosis in LUAD. We also performed survival analysis of ZDHHC5 in 3 molecular subtypes of LUAD (Figure 2H). In the overall survival, high expression of ZDHHC5 only showed lower survival probability in the PP (Proximal-proliferative, formerly magnoid) subgroup (*P* < 0.05), whereas the PI (Proximal-inflammatory, formerly squamoid) and TRU (Terminal respiratory unit, formerly bronchioid) subgroup did not (*P* > 0.05). However, the Kaplan-Meier curves of disease-free survival indicated ZDHHC5 expression was not associated with disease-free survival in the 3 subtypes (*P* > 0.05).

### ZDHHC5 Correlated With Inner Centromere Protein

**FIGURE 3:**
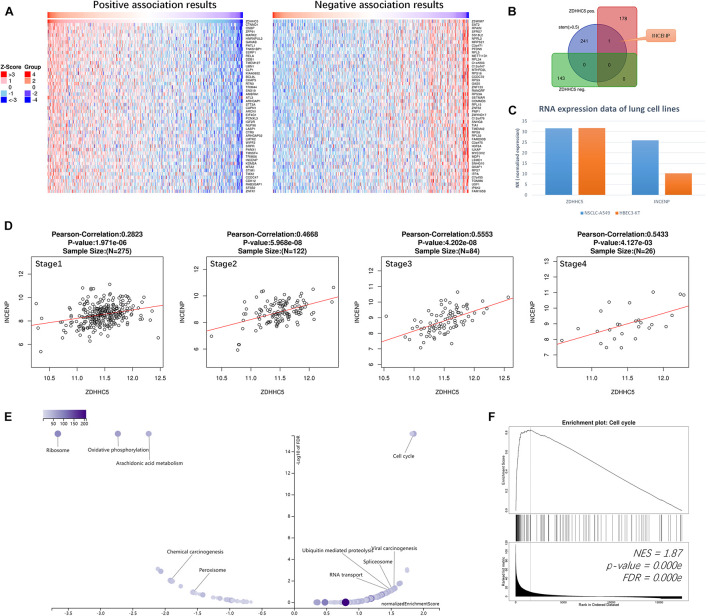
Correlations of ZDHHC5 and INCENP in LUAD. **(A)** Data for the top 50 positive and negative ZDHHC5 association genes are visualized in heat maps. **(B)** Venn diagram showing the mapping results of LUAD stemness genes, ZDHHC5 positive and negative expressed genes. **(C)** The bar plot shows the RNA-seq results of ZDHHC5 and INCENP in A549, HBEC3, and SCLC-21H cell lines from the HPA database. Normalized expression, NX. **(D)** Correlation analysis of ZDHHC5 and INCENP according to the pathological stages of LUAD cases from TCGA, using Pearson’s correlation tests. **(E)** The enriched KEGG pathway result clustered by weighted set cover. In the volcano plots, the intensity of color and jitter size indicates the number of the elements in each pathway. **(F)** GSEA results of cell cycle (hsa04110). NES, normalized enrichment score.

We performed Pearson’s correlation analysis of ZDHHC5 in LUAD. There were 179 positive associations and 143 negative associations (*FDR* < 0.01; *R* > 0.3 for positive, *R* < −0.3 for negative). Heatmaps (Figure 3A) revealed the top 50 positively and negatively associated genes, respectively. ZDHHC5 maintains GSC self-renewal capacity, promotes GSC tumorigenicity, and is essential for preserving the migratory, invasive, and angiogenic potentials of GSCs (Chen et al., 2017). However, the stemness of ZDHHC5 in LUAD has not been reported yet. Previously, we found a set of 242 LUAD CSC genes based on the mRNAsi index (Zhang et al., 2020). We mapped the CSC genes with ZDHHC5 association results, and INCENP was the only intersecting gene (Figure 3B). The INCENP gene expression was up-regulated in LUAD cases and different among LUAD subtypes; the overall survival analysis results indicated an unfavorable prognosis of INCENP in LUAD (Supplementary Figure 2). It has been reported that ZDHHC5 depletion suppresses cell proliferation and colony formation of some NSCLC cell lines but not of human bronchial epithelial cell (HBEC3) (Tian et al., 2015). We analyzed the RNA-seq data of ZDHHC5 and INCENP in the LUAD cell line of A549 and HBEC3. As shown in Figure 3C, the mRNA levels of INCENP decreased in A549 compared with that in HBEC3, but ZDHHC5 did not. The scatter plots of the Pearson’s association analysis results revealed that the correlation between ZDHHC5 and INCENP in LUAD increased by clinical stages (Figure 3D). Next, we generated a set of INCENP correlated genes in LUAD, and used the Stouffer-based meta-analysis method to compare the associations results of ZDHHC5 and INCENP (Supplementary Table 1). Subsequently, we performed Kyoto Encyclopedia of Genes and Genomes (KEGG) pathway enrichment analysis using the results from the meta-analysis using gene set enrichment analysis (GSEA). To reduce redundancy, enriched gene sets were post-processed by the method of Weighted set cover (Vasaikar et al., 2018). Among the clustered results (Figure 3E), the cell cycle pathway (hsa04110) had the highest normalized enrichment score (NES) among the GSEA results (Figure 3F).

### Palmitoylation and Function of Inner Centromere Protein

**FIGURE 4:**
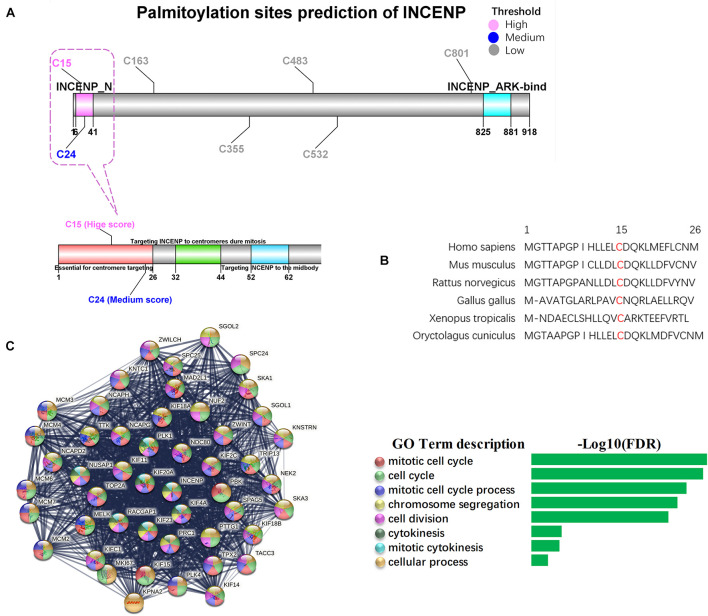
Palmitoylation sites of INCENP and its function in cancer stem cell. **(A)** The palmitoylation sites prediction for INCENP was performed by CCS-Palm 4.0, with the INCENP protein sequence. Cysteine, Cys, C. The color presents high (pink), medium (blue) and low (blue) palmitoylation score, respectively. The significant domains were marked pink (6-41, INCENP_N), and blue (825-881, INCENP ARK-bind). The region surrounding Cys^15^ is shown in detail (bottom). **(B)** The first 26 amino acid sequences of the INCENP across species. **(C)** PPI networks of LUAD stemness genes having direct interaction with INCENP.

As the Pearson’s correlation revealed only the potential physical association between gene expression levels rather than the direct analysis of the interplay between two proteins, we investigated the potential palmitoylation with the amino acid sequences of the two proteins. INCENP can bind to the oncogenes survivin and borealin through its N-terminal domain (INCENP_N) form a trimer to exert its cancer-promoting function (Klein et al., 2006; Vader et al., 2006). The conserved motif encompassing amino acids 32–44 has been proven to be essential for targeting INCENP to centromeres during mitosis, and the first 26 amino acids in the protein sequence of INCENP has also been reported to facilitate this functional targeting centromeres (Ainsztein et al., 1998). However, effects of the deletion of the INCENP protein structure have not been excluded. CSS-Palm 4.0 is a software for *in silico* predicting palmitoylation sites based on proteomic analysis, including a fourth-generation Group-based Prediction System (GPS) algorithm (Ren et al., 2008). The latest training data set contains 583 palmitoylation sites from 277 proteins, experimentally proved and reported in scientific literature. Particle Swarm Optimize (PSO) was also integrated to improve the convergence speed and training accuracy. The prediction performance and system robustness of CSS-Palm 4.0 was evaluated by the leave-one-out validation and 4-, 6-, 8-, 10-fold cross-validations. To further uncover the mechanism underlying the association between INCENP and ZDHHC5 expression, we analyzed the S-palmitoylation sites of INCENP using CSS-Palm 4.0. As shown in Figure 4A, INCENP has 7 cysteine sites predicted to be S-palmitoylated. Among them, Cys^15^ had the highest prediction score of 23, and the scores of other sites were less than 8. Actually, the first 26 amino acids are a non-conserved sequence. This peculiarity makes it difficult to predict its effect on protein structure. In this regard, we analyzed the Cys^15^ site, and found that it was a conserved amino acid site among species, as illustrated in Figure 4B. Taken together, the results indicated that Cys^15^ may be the key site for palmitoylation of INCENP, as well as for targeting centromeres.

To confirm the function of INCENP in CSCs, we extracted genes that were directly associated with INCENP in the LUAD stem cell gene set. Then we constructed a protein-protein interaction (PPI) interaction network using STRING, and conducted a Biological Process (Gene Oncology, GO) enrichment analysis (Figure 4C). The well-known function of INCENP is associated with centromeres, which was responsible for many kinetochore functions, including microtubule binding, motor activity, and cell cycle signaling (Ainsztein et al., 1998). The enrichment analysis results also indicated that the role of INCENP in CSCs was mainly attributable to the cell cycle.

### Subcellular Localization of ZDHHCs Focused Particularly on the Nucleus

**FIGURE 5:**
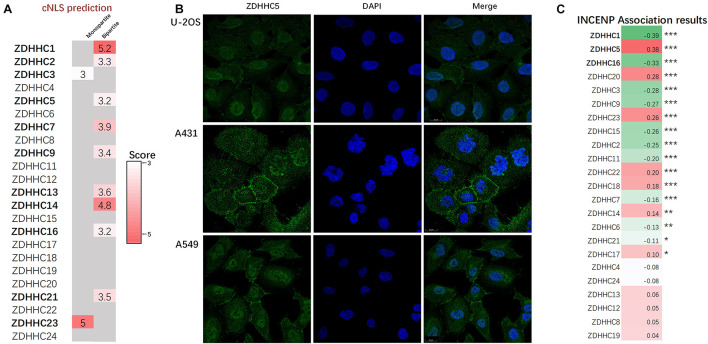
ZDHHC5 is the only nuclear ZDHHC family member correlated with INCENP. **(A)** Results of ZDHHCs nuclear localization signals (NLSs) are shown in the heatmap, calculated by cNLS Mapper. The cNLS Mapper scans the protein sequence with a window size of 16 amino acid residues for monopartite NLSs and 26–28 amino acid residues for bipartite NLSs. **(B)** Immunofluorescence images (HPA) show the subcellular localization of the nuclear ZDHHC proteins (green) with reference DAPI (blue) for the nucleus in cell lines. **(C)** Pearson correlation analysis of INCENP and ZDHHCs in the LUAD. The color represents the corresponding correlation value. Red, positive association; green, negative association. |*Pearson coefficient*| > 0.3 and *P* < 0.05 is significant. *P < 0.05, **P < 0.01, ***P < 0.001.

Since the proteins of the ZDHHCs family all have the function of catalyzing palmitoylation, and ZDHHC5 is not the only ZDHHC that is highly expressed in LUAD, we asked why only ZDHHC5 was associated with poor prognosis in LUAD. It is widely established that palmitoylated proteins are (almost) always localized at the membrane (except ZDHHC23) and palmitoylation influences protein trafficking between the cytomembrane and the cytosol (or Golgi apparatus). However, over the years, several nuclear proteins have been identified as targets of palmitoylation. Further, INCENP is also a nuclear protein, which suggests a potential role for ZDHHC5 in the control of INCENP in the nucleus. In order to determine the subcellular localization of ZDHHCs, we predicted the ZDHHCs nuclear localization signals (NLSs) specific to the importin αβ pathway using cNLS Mapper (Figure 5A). Briefly, a GUS-GFP reporter protein fused to an NLS with a score of 8, 9, or 10 indicates exclusive localization to the nucleus; scores of 7 or 8 indicate partial localization to the nucleus; a score of 3, 4, or 5 indicates localization to both the nucleus and the cytoplasm; and scores of 1 or 2 localization to the cytoplasm. We identified 11 ZDHHCs predicted to be localized in the nuclear compartment and the cytoplasm (scores between 3 and 5). To verify these *in silico* results, we obtained immunofluorescence images of 20 ZDHHC proteins in human cell lines from the HPA database (Supplementary Table 2). We found that ZDHHC5, ZDHHC8, ZDHHC12, ZDHHC14, ZDHHC15, ZDHHC16, and ZDHHC23 localized in the nucleus (Supplementary Figure 3). Figure 5B showed that ZDHHC5 was mainly localized to the plasma membrane and nucleoplasm in A549, in A431 (an epidermoid carcinoma cell line), and in U-2 OS (an osteosarcoma cell line). There were 4 ZDHHCs predicted and verified to be located in nucleus, including ZDHHC5, ZDHHC14, ZDHHC16, and ZDHHC23.

Since ZDHHC5 is not the only ZDHHC protein with nuclear localization, we also performed correlation analysis to define the specificity of the effects of ZDHHC5 and INCENP (Figure 5C). The Pearson’s correlation analysis showed that ZDHHC5 was the only ZDHHC protein positively correlated with INCENP gene expression. Although ZDHHC16 also showed nuclear localization, its gene expression was negatively associated with INCENP. Accumulating evidence has indicated that the nuclear translocation of ZDHHC5 plays an important role in the regulation of many biological processes including S-palmitoylation, and its down-regulation is almost certainly involved in LUAD tumorigenesis.

## Discussion

Protein S-palmitoylation plays an essential role in the function of both oncogenes and tumor suppressors (Resh, 2017). S-palmitoylation is mediated by a family of 23 members encoded by ZDHHC genes. An increasing number of studies have indicated that dysfunction and/or dysregulation of the ZDHHC proteins are important in tumorigenesis, such as ovarian cancer (Yuan et al., 2020), glioma (Chen et al., 2017, 2020b; Codony-Servat et al., 2019), and kidney renal clear cell carcinoma (Liu et al., 2020). In lung cancer, the screening of ZDHHCs has been performed in cell lines, but has not been evaluated in tissues from patients (Tian et al., 2015). Herein, we used high-throughput data of LUAD patients to identify key ZDHHCs from the ZDHHC family of palmitoyl S-acyltransferases. The data source including RNA-seq data of TCGA, microarray series from GEO, and mass spectrometry-based proteomic data generated by CPTAC. Among the differential expression ZDHHCs, ZDHHC5 was the only gene with prognostic value.

We found that ZDHHC5 was overexpressed in LUAD patients, which was based on large sample size. However, high-throughput data are challenging in the regard of reproducibility, so *in vivo* or *in vitro* experiments could further confirm our findings. Tian’s team has chosen 3 HBEC lines and 11 NSCLC cell lines, including the LUAD subtype, to explore the differential expression of ZDHHC5. The western blot revealed the upregulation of ZDHHC5 protein expression in NSCLC cell lines (Tian et al., 2015), which supported our finding. The biological function of ZDHHC5 in LUAD has been explored *in vitro*. Knocking-down ZDHHC5 inhibited the cell proliferation and colony formation of NSCLC cell lines, but not HBECs (Tian et al., 2015).

CSCs participate in cancer immunosurveillance and immune evasion through a variety of mechanisms (Maccalli et al., 2018). Across TCGA cancer types, Thorsson et al. (2018) identified six immune subtypes characterized by differences in macrophage or lymphocyte signatures, Th1:Th2 cell ratio, intratumoral heterogeneity, aneuploidy, the extent of neoantigen load, overall cell proliferation, expression of immunomodulatory genes, and prognosis. Therefore, we investigated ZDHHC5 for its potential association with tumor immunity and possible mechanism. In this study, we analyzed ZDHHC5 expression in immune subtypes and the correlation with the tumor microenvironment of LUAD. The results show that the gene expression of ZDHHC5 distributes evenly among immune subtypes, and we found the expression of ZDHHC5 was not associated with the abundance of immunomodulators and tumor-infiltrating lymphocytes. These results indicated that ZDHHC5 might not be involved in the mechanism of immunotherapy in LUAD.

The new nomenclature for LUAD molecular subtype is a key step in lung cancer diagnosis and treatment. The molecular classification is characterized by specific genetic alterations, including histopathological, anatomic, and mutational categorizations. Analysis of the TCGA transcriptional profiling data has provided 3 molecular subtypes of LUAD. (1) The PI subtype was characterized by the Solid architecture with enrichment for NF1 and TP53 in addition to p16 methylation. (2) The PP subtype was enriched for mutations and CNAs in KRAS and STK11, along with variable histology. (3) The TRU subtype harbored mutations or CNAs in EGFR and kinase fusions. Among the three molecular subtypes, TRU has the most favorable prognosis. The TRU subtype presents more commonly in women who never smoked, characterized by Acinar, Papillary, or Lepidic histomorphology. In this work, the gene expression level of ZDHHC5 is highest in PI, relatively low in TRU, and lowest in PP (Supplementary Figure 1B). Although the result is not statistically significant, we still attribute it to the cancer-promoting effect of ZDHHC5, considering the small sample size.

ZDHHC5 has been reported to mediate palmitoylation in TP53-mutant gliomas and drives malignant development and progression (Chen et al., 2017). Similarly, ZDHHC5 expression was increased in the TP53 mutant LUAD group (Figure 2D). As TP53 mutation is one of the PI’s molecular characters, this result consists with the high expression of ZDHHC5 in PI subtypes. Therapies targeting the palmitoylation process may be a potential strategy in treatments of TP53 mutation LUAD patients.

The TRU subtype associates with gender and smoking habits. However, the expression of ZDHHC5 in LUAD patients of different gender and smoking habits was not significantly different (Supplementary Figures 1C,D). Another character of TRU is the histomorphological feature of acinar, papillary, or lepidic, and these subtypes were associated with a good prognosis. In this study, the clinical pathologist of our group scored ZDHHC5 stained in the IHC samples according to the histomorphology classification (Travis et al., 2015). Unfortunately, due to the relatively small number of LUAD samples in the HPA database (*n* = 12), we can only confirm that ZDHHC5 can express in the 5 pathological types of Solid, Micropapillary, Acinar, Lepidic, and Enteric, but cannot conclude the prognostic value of ZDHHC5 based on these scores. The above results suggest that although ZDHHC5 gene expression is not associated with gender and smoking, the prognostic value of ZDHHC5 expression in different pathological subtypes should be explored in larger cohorts for future studies.

Another distinct finding of this study was the identification of the prognostic value of ZDHHC5 in LUAD, in contrast to that reported by Tian et al. (2015). Tian et al. (2015) performed survival analysis in a NSCLC cohort of 194 patients according to ZDHHC5 expression by immunostaining, and reported that the differences in expression were not statistically significant. In this study, we performed survival analysis of ZDHHC5 in 17 independent LUAD cohorts comprising 2244 cases. Meta-analysis was used to avoid biases and misinterpretations. Furthermore, a solid understanding of the IHC contrasting mechanism also positively impacted on determining the outcomes of the staining protocol. In fact, only membrane staining of ZDHHC5, without nuclear staining, was scored in Tian et al. (2015)’s study. In addition, NSCLC is comprised of several histological subtypes, and the expression of ZDHHC5 differs across different subtypes. Thus, we focused on the prognostic value of ZDHHC5 within adenocarcinoma, which should also be prospectively addressed.

Although ZDHHC5 or palmitoylation has been linked to lung cancer (Tian et al., 2015; Ali et al., 2018), its association with CSC has not been reported. Earlier, we analyzed a gene-set including hundreds of novel CSC biomarkers based on mRNAsi index and profiled a network of LUAD stem cell genes. We then asked whether these CSC genes could be palmitoylated. To this aim, our initial approach involved a correlation analysis and S-palmitoylation site prediction evaluation. By mapping ZDHHC5 correlated genes and mRNAsi-based cancer stemness genes, we identified INCENP, a stemness gene, as the bridge between ZDHHC5 and CSCs in LUAD.

The N-terminal domain (INCENP_N) is required for chromosomal passenger complex localization to centromeres (Ainsztein et al., 1998) and forms a three-helix bundle with borealin and survivin (Klein et al., 2006; Vader et al., 2006). Ainsztein et al. (1998) have suggested that the conserved motif encompassing amino acids 32–44 is essential for targeting INCENP to centromeres during mitosis. Since the sequence of aa 1-26 is not conserved among species, they prepared a deletion construct (INCENP 27-839) encoding a protein missing the first 26 amino acids. However, the INCENP 27-839 failed to target centromeres. Unfortunately, they did not conduct in-depth research on the aa 1-26 of INCENP, only attributing the effect to INCENP protein structure (Ainsztein et al., 1998). In our study, we suppose Cys^15^ as an S-palmitoylation site of INCENP. By analyzing the amino acid sequence of INCENP *in silico*, we found Cys^15^ had the highest S-palmitoylation score. Surprisingly, Cys^15^ is a consensus amino acid site among species (Figure 4B). Such conservation indicates that Cys^15^ may serve a critical biological function. Taken together, the S-palmitoylation of Cys^15^ may serve as a potential mechanism to explain the function of aa 1-26 in the INCENP protein sequence. We suggest that the function of ZDHHC5 to promote cell proliferation is achieved by regulating the palmitoylation status of its substrate. INCENP protein is a potential substrate of ZDHHC5. In our study, RNA-seq data showed that INCENP was upregulated in LUAD cell line of A549, compared to HBEC3 (Figure 3C). The IHC staining intensity and density of INCENP protein in NSCLC tissues (*n* = 104) were significantly higher than those in paired adjacent normal lung tissues (Xia et al., 2015). Therefore, due to the low expression level of INCENP in HBEC3, the decline of palmitoylation has no significant effect on cell proliferation.

Protein palmitoylation increases the protein hydrophobicity by lipid modification in cancer cells, facilitating the proteins’ propensity to anchor to any membrane. To date, the palmitoylome has been mainly described in the cytosol and the Golgi apparatus. INCENP is a nucleoprotein, which plays a critical role at the centromere in ensuring strict chromosome alignment and segregation during mitosis (Klein et al., 2006; Becker et al., 2010). The reversible S-palmitoylation of INCENP may not only take place in the cytoplasm but may also occur in the nucleus. We unexpectedly discovered that ZDHHC5, ZDHHC14, ZDHHC16, and ZDHHC23 were located in the cell nucleus in our analysis. This localization may facilitate the propensity of INCENP proteins to anchor to nuclear membranes and reach the nucleus; although, we may consider its nuclear location as a potential prerequisite for nucleoprotein palmitoylation within the nucleus. Thus, although the evaluation of the direct interaction between ZDHHC5 and INCENP was not sufficiently performed in this study, its nuclear localization and the presence of the S-palmitoylation site appear to be essential for timely, complete, and efficient S-palmitoylation to occur.

## Conclusion

This study identified ZDHHC5 as a key PAT in LUAD for its over-expression and malignant prognosis in LUAD patients, overturning its previously refuted prognostic value. As digging the stemness substrate of ZDHHC5, we found INCENP was correlated with the gene expression of ZDHHC5 in LUAD. Importantly, the analysis of the S-palmitoylation site brings us to speculation that ZDHHC5 catalyzes the S-palmitoylation at Cys^15^ of INCENP. By analyzing the nuclear localization signals and observing immunofluorescence results, we found that ZDHHC5 locates in the nucleoplasm, in addition to the plasma membrane. Our work highlights the essential role of ZDHHC5 in LUAD and reveals that ZDHHC5 modulates the S-palmitoylation of INCENP. However, further research including *in vivo* and *in vitro* experiments is needed to validate ZDHHC5 as a molecular target in the therapy of LUAD patients.

## Materials and Methods

### Database

The RNA-seq results and clinical data for LUAD patients (*n* = 515) and normal samples (*n* = 59) was obtained from the LUAD dataset of the TCGA database.^1^ The GEO database^2^ is an open-access platform, providing gene expression results performed by microarray, including clinical data. The CPTAC is a database, designed for large-scale proteome and genome analysis. Protein expression analysis in this study used mass-spectrometry-based proteomic data from the CPTAC Confirmatory/Discovery cohorts. The HPA (Uhlén et al., 2015) is an open access knowledge resource, with the aim to map all the human proteins in cells, tissues and organs. The HPA consists of six separate parts, and the Tissue Atlas (Uhlén et al., 2005), Pathology Atlas (Uhlen et al., 2017), Cell Type Atlas and Cell Atlas (Thul et al., 2017) were used in this study.

### Differential Expression Analysis

UALCAN^3^ is a web resource providing open access to TCGA and CPTAC data. We performed mRNA and protein gene expression analysis in UALCAN. RNA-seq results of mRNA expression was normalized as Transcript per million (TPM), and the expression data is *log_2_ (TPM* + *1)* transformed. As there was an observed high variability in within-profile expression median or standard deviation across samples, the log2 spectral count ratio values from CPTAC were first normalized to standard deviations from the median value within each sample profile, and then normalized across the sample. *Z*-values represent standard deviations from the median across samples for LUAD. We used the median to calculate the deviation between LUAD and the normal cases. Significant differences were estimated by Student’s *t*-test, and a *P*-value < 0.05 was considered to define differentially expressed genes (DEGs).

The Single Cell Type Atlas, stores expression data of protein-coding genes in single human cell types. Normalized RNA expression (NX) data of lung cell lines was analyzed by the Cell Type Atlas of the HPA and visualized in bar plot.

### Survival Analysis

The survival map and Kaplan-Meier curve were generated from Gene Expression Profiling and Interactive Analyses vision 2 (GEPIA2).^4^ The OS or DFS analysis was based on gene expression and survival time; the median was selected as the threshold for splitting the high-expression and low-expression cohorts (Cut-off = 50%); the HR based on Cox proportional hazards Model were calculated; *P-*value < 0.05 was considered statistically significant using the log-rank test.

The Lung Cancer Explorer (LCE) (Cai et al., 2019) is an open access web resource, which collects data from 56 lung cancer studies that include over 6700 patients. In this study, LCE was employed to evaluate the prognostic value of ZDHHC5 and INCENP using meta-analysis. Forest plots generated by the LCE meta-analysis module indicate heterogeneity test using the *I*^2^ statistic, which refers to the percentage of variation across datasets that is due to heterogeneity. All data was obtained from 19 independent LUAD studies from GEO and TCGA, listed as Tomida_2009 (GSE13213), Hou_2010 (GSE19188), Wikerson_2012 (GSE17710), Staaf_2012 (GSE29016), Kuner_2009 (GSE10245), Rousseaux_2013 (GSE30219), Okayama_2012 (GSE31210), Bild_2006 (GSE3141), Girard_N_c (GSE31548), Botling_2013 (GSE37745), Jones_2004 (GSE1037), Sato_2013 (GSE41271), Tang_2013 (GSE42127), Der_2014 (GSE50081), Schabath_2016 (GSE72094), Zhu_2010 (GSE14814), Takeuchi_2006 (GSE11969), Shedden_2008 (Consortium) (Shedden et al., 2008), and TCGA_LUAD_2016. In this study, *I*^2^ = 45% (ZDHHC5) and 54% (INCENP), which indicates intermediate different heterogeneity of LUAD datasets.

### Immune Analysis

The Tumor–Immune System Interactions Data Base (TISIDB) is an integrated repository portal for tumor and immune system interaction (Ru et al., 2019). Distribution of gene expression across immune subtypes (Thorsson et al., 2018) using TCGA data was also analyzed in TISIDB.

### Correlation Analysis

The *LinkFinder* module of LinkedOmics^5^ (Vasaikar et al., 2018) was used to search and visualize genes associated with ZDHHC5 and INCENP mRNA expression in LUAD cases from the TCGA database (515 patients). The results were analyzed using Pearson’s correlation analysis. R represents the Pearson correlation coefficient. Genes with the result of | *R* | > 0.3 and *FDR* < 0.05 were considered statistically significant.

### Immunohistochemistry

The HPA contains images of histological sections from normal (Tissue Atlas) and cancer tissues (Human Pathology Atlas) obtained by IHC. Since specimens are derived from surgical material, normal was defined as non-neoplastic and morphologically normal. Tissue microarrays were used to show ZDHHC5 antibody (Atlas Antibodies Cat#HPA014670, RRID:AB_2257442) staining in samples from 3 individuals defined as normal lung tissue, and samples from 6 cancer patients corresponding to 12 LUAD samples. Each sample was represented by 1-mm tissue cores. Basic annotation parameters included an evaluation of (i) staining intensity (negative, weak, moderate, or strong), (ii) fraction of stained cells (<25, 25–75, or > 75%), and iii) subcellular localization (nuclear and/or cytoplasmic/membranous). The histological subtype was classified by a clinical pathologist in our group, and semiquantitative estimation of the different histological patterns present in 5% increments according to the 2015 WHO Classification of Lung Tumors (Travis et al., 2015). LUAD is classified according to the most representative pattern in the cross-sectional area of the tissue section (the so-called main pattern), and pathological types are introduced according to the main pattern (Kuhn et al., 2018).

### Immunofluorescence and Confocal Microscopy

The Cell Atlas of the HPA database displays high-resolution, multicolor images of proteins labeled by indirect immunofluorescence. The standard immunostaining protocol for immunofluorescence can be found in the open access repository for science methods at protocols. The cells were stained with the HPA antibody of ZDHHCs protein (green) and DAPI for the nucleus (blue).

### Prediction of S-Palmitoylation Sites and Nuclear Localization Signals

The prediction of S-palmitoylation sites in INCENP was performed by GPS-Palm (*CSS-Palm 4.0*), a deep learning-based graphic presentation system for the prediction of S-palmitoylation sites in proteins. We used InterPro to predict domains and important sites of INCENP.

We predicted the ZDHHCs nuclear localization signals (NLSs) specific to the importin αβ pathway by cNLS Mapper, based on the amino acid sequence of each protein. cNLS Mapper extracted the putative NLS sequences with a score equal to or more than the selected cut-off score. Higher scores indicated stronger NLS activities.

### Construction of the Protein-Protein Interaction Network

The PPI network was retrieved from STRING v.11.0.^6^ The minimum required interaction score was set at medium confidence (0.400). Active interaction sources included text-mining, experiments, databases, co-expression, neighborhood, gene fusion, and co-occurrence. Only individual networks with 10 or more nodes were included for further analysis. Disconnected nodes were hidden in the network. The edges indicated both functional and physical protein associations. Each node represented an input protein, and the line thickness indicated the strength of data support.

### Enrichment Analysis

We performed Gene Oncology (GO) functional enrichment with genes in each PPI network using STRING. FDR indicated *P*-values corrected for multiple testing within each category using the Benjamini-Hochberg procedure. FDR was log_10_ transformed to describe the significance of the enrichment and visualized in a bar-plots according to the BP, biological process; MF, molecular function; and CC, cellular component.

The *Link-Interpreter* module of LinkedOmics was used to perform enrichment analysis of the meta-analysis results of ZDHHC5 and INCENP associated genes. In the *Link-Interpreter* module, the meta data was signed and ranked by the meta *P*-value, and Gene Set Enrichment Analysis (GSEA) (Subramanian et al., 2005) was used to generate analyses based on the Kyoto Encyclopedia of Genes and Genomes (KEGG) pathway. The minimum number within per gene size was set as 5, and 1,000 simulations were performed.

### Stemness Feature Analysis

The mRNA expression-based stemness index (mRNAsi index) (Malta et al., 2018) was used to assess the degree of oncogenic dedifferentiation, which was calculated using an innovative OCLR machine-learning algorithm (Sokolov et al., 2016). Higher mRNAsi scores were associated with malignant biological processes in CSCs and greater tumor dedifferentiation, according to the histopathological grades. In our previous study (Ruiz-Cordero and Devine, 2020), we calculated a set of LUAD stemness genes based on the mRNAsi index. Using WGCNA, stemness genes were clustered into modules. Key genes were screened from the blue module based on the gene significance for mRNA stemness indices (GS.mRNAsi), gene significance for the epigenetically regulated-mRNAsi (GS EREG-mRNAsi) and module membership (MM) scores. In this study, we used genes with the GS mRNAsi/GS EREG-mRNAsi score > 0.5 and *P* < 0.05 as stem genes for further analysis.

## Data Availability Statement

Publicly available datasets were analyzed in this study. This data can be found here: https://portal.gdc.cancer.gov/, https://www.ncbi.nlm.nih.gov/geo/, and https://cptac-data-portal.georgetown.edu/datasets.

## Author Contributions

HL and FL: conception and design. XL: administrative support. YZ: collection and assembly of data. YZ, KF, XL, FL, and I-CL: data analysis and interpretation. All authors manuscript writing and final approval of manuscript.

## Conflict of Interest

I-CL was employed by the company Insight Genomics Inc. The remaining authors declare that the research was conducted in the absence of any commercial or financial relationships that could be construed as a potential conflict of interest.

## Publisher’s Note

All claims expressed in this article are solely those of the authors and do not necessarily represent those of their affiliated organizations, or those of the publisher, the editors and the reviewers. Any product that may be evaluated in this article, or claim that may be made by its manufacturer, is not guaranteed or endorsed by the publisher.

## Footnotes

1
https://portal.gdc.cancer.gov/

2
https://www.ncbi.nlm.nih.gov/geo/

3
http://ualcan.path.uab.edu

4
http://gepia2.cancer-pku.cn/

5
http://www.linkedomics.org/login.php

6
https://string-db.org/

## Abbreviations

CPTAC: Clinical Proteomic Tumor Analysis Consortium
CSC: Cancer stem cells
DFS: disease free survival
EGFR: epidermal growth factor receptor
EREG-mRNAsi: RNA expression-based Epigenetically regulated-mRNAsi
GEO: Gene Expression Omnibus
GPS: Group-based Prediction System
GO: Gene Oncology
GS: gene significance
GSC: glioma stem cell
GSEA: gene set enrichment analysis
HBEC: human bronchial epithelial cell
HPA: Human Protein Atlas
HR: hazard ratio
INCENP: inner centromere protein
KEGG: Kyoto Encyclopedia of Genes and Genomes
LUAD: lung adenocarcinoma
MM: module membership
MS: mass spectrometry
mRNAsi: mRNA stemness indices
NES: normalized enrichment score
NLS: nuclear localization signal
NSCLC: non-small cell lung cancer
NX: Normalized expression
OCLR: one-class logistic regression
OS: overall survival
PPI: protein-protein interaction
PSO: Particle Swarm Optimize
TCGA: The Cancer Genome Atlas
TKI: tyrosine kinase inhibitor
TPM: Transcript per million
WGCNA: weighted gene co-expression network analysis
WHO: World Health Organization
ZDHHC: zinc finger Asp-His-His-Cys.

## References

[B1] Ainsztein A. M. Kandels-Lewis S. E. Mackay A. M. Earnshaw W. C. (1998). INCENP centromere and spindle targeting: identification of essential conserved motifs and involvement of heterochromatin protein HP1. *J. Cell Biol.* 143 1763–1774. 10.1083/jcb.143.7.1763 9864353 PMC2175214

[B2] Ali A. Levantini E. Teo J. T. Goggi J. Clohessy J. G. Wu C. S. (2018). Fatty acid synthase mediates EGFR palmitoylation in EGFR mutated non-small cell lung cancer. *EMBO Mol. Med.* 10:e8313. 10.15252/emmm.201708313 29449326 PMC5840543

[B3] Becker M. Stolz A. Ertych N. Bastians H. (2010). Centromere localization of INCENP-Aurora B is sufficient to support spindle checkpoint function. *Cell Cycle* 9 1360–1372. 10.4161/cc.9.7.11177 20372054

[B4] Cai L. Lin S. Girard L. Zhou Y. Yang L. Ci B. (2019). LCE: an open web portal to explore gene expression and clinical associations in lung cancer. *Oncogene* 38 2551–2564. 10.1038/s41388-018-0588-2 30532070 PMC6477796

[B5] Cancer Genome Atlas Research Network (2014). Comprehensive molecular profiling of lung adenocarcinoma. *Nature* 511 543–550. 10.1038/nature13385 25079552 PMC4231481

[B6] Chan P. Han X. Zheng B. DeRan M. Yu J. Jarugumilli G. K. (2016). Autopalmitoylation of TEAD proteins regulates transcriptional output of the Hippo pathway. *Nat. Chem. Biol.* 12 282–289. 10.1038/nchembio.2036 26900866 PMC4798901

[B7] Chen X. Hao A. Li X. Ye K. Zhao C. Yang H. (2020a). Activation of JNK and p38 MAPK Mediated by ZDHHC17 Drives Glioblastoma Multiforme Development and Malignant Progression. *Theranostics* 10 998–1015. 10.7150/thno.40076 31938047 PMC6956818

[B8] Chen X. Hu L. Yang H. Ma H. Ye K. Zhao C. (2019). DHHC protein family targets different subsets of glioma stem cells in specific niches. *J. Exp. Clin. Cancer Res.* 38:25. 10.1186/s13046-019-1033-2 30658672 PMC6339410

[B9] Chen X. Li H. Fan X. Zhao C. Ye K. Zhao Z. (2020b). Protein Palmitoylation Regulates Cell Survival by Modulating XBP1 Activity in Glioblastoma Multiforme. *Mol. Ther. Oncolytics* 17 518–530. 10.1016/j.omto.2020.05.007 33024813 PMC7525067

[B10] Chen X. Ma H. Wang Z. Zhang S. Yang H. Fang Z. (2017). EZH2 Palmitoylation Mediated by ZDHHC5 in p53-Mutant Glioma Drives Malignant Development and Progression. *Cancer Res.* 77 4998–5010. 10.1158/0008-5472.CAN-17-1139 28775165

[B11] Codony-Servat J. Codony-Servat C. Cardona A. F. Giménez-Capitán A. Drozdowskyj A. Berenguer J. (2019). Cancer Stem Cell Biomarkers in EGFR-Mutation-Positive Non-Small-Cell Lung Cancer. *Clin. Lung Cancer* 20 167–177. 10.1016/j.cllc.2019.02.005 30885551

[B12] Dunphy J. T. Linder M. E. (1998). Signalling functions of protein palmitoylation. *Biochim. Biophys. Acta* 1436 245–261. 10.1016/s0005-2760(98)00130-1 9838145

[B13] Fukata Y. Bredt D. S. Fukata M. (2006). “Protein Palmitoylation by DHHC Protein Family,” in *The Dynamic Synapse: Molecular Methods in Ionotropic Receptor Biology*, eds Kittler J. T. Moss S. J. (Boca Raton: CRC Press), 83–88.

[B14] Jiang H. Zhang X. Chen X. Aramsangtienchai P. Tong Z. Lin H. (2018). Protein lipidation: occurrence, mechanisms, biological functions, and enabling technologies. *Chem. Rev.* 118 919–988. 10.1021/acs.chemrev.6b00750 29292991 PMC5985209

[B15] Klein U. R. Nigg E. A. Gruneberg U. (2006). Centromere targeting of the chromosomal passenger complex requires a ternary subcomplex of Borealin, Survivin, and the N-terminal domain of INCENP. *Mol. Biol. Cell* 17 2547–2558. 10.1091/mbc.e05-12-1133 16571674 PMC1474794

[B16] Ko P. J. Dixon S. J. (2018). Protein palmitoylation and cancer. *EMBO Rep.* 19:e46666. 10.15252/embr.201846666 30232163 PMC6172454

[B17] Kuhn E. Morbini P. Cancellieri A. Damiani S. Cavazza A. Comin C. E. (2018). Adenocarcinoma classification: patterns and prognosis. *Pathologica* 110 5–11. 30259909

[B18] Lanyon-Hogg T. Faronato M. Serwa R. A. Tate E. W. (2017). Dynamic protein acylation: new substrates, mechanisms, and drug targets. *Trends Biochem. Sci.* 42 566–581. 10.1016/j.tibs.2017.04.004 28602500

[B19] Leão R. Domingos C. Figueiredo A. Hamilton R. Tabori U. Castelo-Branco P. (2017). Cancer Stem Cells in Prostate Cancer: implications for Targeted Therapy. *Urol. Int.* 99 125–136. 10.1159/000455160 28142149

[B20] Li Y. Martin B. R. Cravatt B. F. Hofmann S. L. (2012). DHHC5 protein palmitoylates flotillin-2 and is rapidly degraded on induction of neuronal differentiation in cultured cells. *J. Biol. Chem.* 287 523–530. 10.1074/jbc.M111.306183 22081607 PMC3249106

[B21] Liu Z. Liu C. Xiao M. Han Y. Zhang S. Xu B. (2020). Bioinformatics Analysis of the Prognostic and Biological Significance of ZDHHC-Protein Acyltransferases in Kidney Renal Clear Cell Carcinoma. *Front. Oncol.* 10:565414. 10.3389/fonc.2020.565414 33364189 PMC7753182

[B22] Lortet-Tieulent J. Soerjomataram I. Ferlay J. Rutherford M. Weiderpass E. Bray F. (2014). International trends in lung cancer incidence by histological subtype: adenocarcinoma stabilizing in men but still increasing in women. *Lung Cancer* 84 13–22. 10.1016/j.lungcan.2014.01.009 24524818

[B23] Maccalli C. Rasul K. I. Elawad M. Ferrone S. (2018). The role of cancer stem cells in the modulation of anti-tumor immune responses. *Semin. Cancer Biol.* 53 189–200. 10.1016/j.semcancer.2018.09.006 30261276 PMC8668198

[B24] Malta T. M. Sokolov A. Gentles A. J. Burzykowski T. Poisson L. Weinstein J. N. (2018). Machine Learning Identifies Stemness Features Associated with Oncogenic Dedifferentiation. *Cell* 173 338–354.e15. 10.1016/j.cell.2018.03.034 29625051 PMC5902191

[B25] Miyata T. Yoshimatsu T. So T. Oyama T. Uramoto H. Osaki T. (2015). Cancer stem cell markers in lung cancer. *Pers. Med. Univ.* 4 40–45. 10.1016/j.pmu.2015.03.007

[B26] Percherancier Y. Planchenault T. Valenzuela-Fernandez A. Virelizier J. L. Arenzana-Seisdedos F. Bachelerie F. (2001). Palmitoylation-dependent control of degradation, life span, and membrane expression of the CCR5 receptor. *J. Biol. Chem.* 276 31936–31944. 10.1074/jbc.M104013200 11390405

[B27] Ren J. Wen L. Gao X. Jin C. Xue Y. Yao X. (2008). CSS-Palm 2.0: an updated software for palmitoylation sites prediction. *Protein Eng. Des. Sel.* 21 639–644. 10.1093/protein/gzn039 18753194 PMC2569006

[B28] Resh M. D. (2017). Palmitoylation of proteins in cancer. *Biochem. Soc. Trans.* 45 409–416. 10.1042/BST20160233 28408481

[B29] Riccio A. (2010). Dynamic epigenetic regulation in neurons: enzymes, stimuli and signaling pathways. *Nat. Neurosci.* 13 1330–1337. 10.1038/nn.2671 20975757

[B30] Ru B. Wong C. N. Tong Y. Zhong J. Y. Zhong S. S. W. Wu W. C. (2019). TISIDB: an integrated repository portal for tumor-immune system interactions. *Bioinformatics* 35 4200–4202. 10.1093/bioinformatics/btz210 30903160

[B31] Ruiz-Cordero R. Devine W. P. (2020). Targeted Therapy and Checkpoint Immunotherapy in Lung Cancer. *Surg. Pathol. Clin.* 13 17–33. 10.1016/j.path.2019.11.002 32005431

[B32] Shedden K. Taylor J. M. Enkemann S. A. Tsao M. S. Yeatman T. J. Gerald W. L. (2008). Gene expression-based survival prediction in lung adenocarcinoma: a multi-site, blinded validation study. *Nat. Med.* 14 822–827. 10.1038/nm.1790 18641660 PMC2667337

[B33] Sokolov A. Paull E. O. Stuart J. M. (2016). ONE-CLASS DETECTION OF CELL STATES IN TUMOR SUBTYPES. *Pac. Symp. Biocomput.* 21 405–416. 10.1142/9789814749411_0037 26776204 PMC4856035

[B34] Spinelli M. Fusco S. Grassi C. (2018). Nutrient-Dependent Changes of Protein Palmitoylation: impact on Nuclear Enzymes and Regulation of Gene Expression. *Int. J. Mol. Sci.* 19:3820. 10.3390/ijms19123820 30513609 PMC6320809

[B35] Subramanian A. Tamayo P. Mootha V. K. Mukherjee S. Ebert B. L. Gillette M. A. (2005). Gene set enrichment analysis: a knowledge-based approach for interpreting genome-wide expression profiles. *Proc. Natl. Acad. Sci. U. S. A.* 102 15545–15550. 10.1073/pnas.0506580102 16199517 PMC1239896

[B36] Thorsson V. Gibbs D. L. Brown S. D. Wolf D. Bortone D. S. Ou Yang T. H. (2018). The Immune Landscape of Cancer. *Immunity* 48 812–830.e14. 10.1016/j.immuni.2018.03.023 29628290 PMC5982584

[B37] Thul P. J. Åkesson L. Wiking M. Mahdessian D. Geladaki A. Ait Blal H. (2017). A subcellular map of the human proteome. *Science* 356:eaal3321. 10.1126/science.aal3321 28495876

[B38] Tian H. Lu J. Y. Shao C. Huffman K. E. Carstens R. M. Larsen J. E. (2015). Systematic siRNA Screen Unmasks NSCLC Growth Dependence by Palmitoyltransferase DHHC5. *Mol. Cancer Res.* 13 784–794. 10.1158/1541-7786 25573953 PMC4398612

[B39] Travis W. D. Brambilla E. Nicholson A. G. Yatabe Y. Austin J. H. M. Beasley M. B. (2015). The 2015 World Health Organization Classification of Lung Tumors: impact of Genetic, Clinical and Radiologic Advances Since the 2004 Classification. *J. Thorac. Oncol.* 10 1243–1260. 10.1097/JTO.0000000000000630 26291008

[B40] Uhlén M. Björling E. Agaton C. Szigyarto C. A. Amini B. Andersen E. (2005). A human protein atlas for normal and cancer tissues based on antibody proteomics. *Mol. Cell Proteomics* 4 1920–1932. 10.1074/mcp.M500279-MCP200 16127175

[B41] Uhlén M. Fagerberg L. Hallström B. M. Lindskog C. Oksvold P. Mardinoglu A. (2015). Proteomics. Tissue-based map of the human proteome. *Science* 347:1260419. 10.1126/science.1260419 25613900

[B42] Uhlen M. Zhang C. Lee S. Sjöstedt E. Fagerberg L. Bidkhori G. (2017). A pathology atlas of the human cancer transcriptome. *Science* 357:eaan2507. 10.1126/science.aan2507 28818916

[B43] Vader G. Kauw J. J. Medema R. H. Lens S. M. (2006). Survivin mediates targeting of the chromosomal passenger complex to the centromere and midbody. *EMBO Rep.* 7 85–92. 10.1038/sj.embor.7400562 16239925 PMC1369225

[B44] Vasaikar S. V. Straub P. Wang J. Zhang B. (2018). LinkedOmics: analyzing multi-omics data within and across 32 cancer types. *Nucleic Acids Res.* 46 D956–D963. 10.1093/nar/gkx1090 29136207 PMC5753188

[B45] Wadowska K. Bil-Lula I. Trembecki Ł Śliwińska-Mossoń M. (2020). Genetic Markers in Lung Cancer Diagnosis: a Review. *Int. J. Mol. Sci.* 21:4569. 10.3390/ijms21134569 32604993 PMC7369725

[B46] Wilson J. P. Raghavan A. S. Yang Y. Y. Charron G. Hang H. C. (2011). Proteomic analysis of fatty-acylated proteins in mammalian cells with chemical reporters reveals S-acylation of histone H3 variants. *Mol. Cell Proteomics* 10:M110.001198. 10.1074/mcp.M110.001198 21076176 PMC3047146

[B47] Xia R. Chen S. Chen Y. Zhang W. Zhu R. Deng A. (2015). A chromosomal passenger complex protein signature model predicts poor prognosis for non-small-cell lung cancer. *Onco Targets Ther.* 8 721–726. 10.2147/OTT.S81328 25897247 PMC4396580

[B48] Yuan M. Chen X. Sun Y. Jiang L. Xia Z. Ye K. (2020). ZDHHC12-mediated claudin-3 S-palmitoylation determines ovarian cancer progression. *Acta Pharm. Sin. B.* 10 1426–1439. 10.1016/j.apsb.2020.03.008 32963941 PMC7488353

[B49] Zhang Y. Tseng J. T. Lien I. C. Li F. Wu W. Li H. (2020). mRNAsi Index: machine Learning in Mining Lung Adenocarcinoma Stem Cell Biomarkers. *Genes* 11:257. 10.3390/genes11030257 32121037 PMC7140876

