# Supplementary material for: Potential Role of S-Palmitoylation in Cancer Stem Cells of Lung Adenocarcinoma
Source: Front Cell Dev Biol. 2021 Sep 21;9:734897. doi: 10.3389/fcell.2021.734897 (PMC8490697; doi:10.3389/fcell.2021.734897)
Supplement: Supplementary file 1 [file Data_Sheet_1.docx]

Supplementary Material

# Supplementary Tables

**Supplementary Table 1.** Comparation of the associations results of ZDHHC5 and INCENP by the Stouffer-based meta-analysis method (provided in a separate Excel file)

**Supplementary Table 2.** Immunofluorescence images of ZDHHC proteins in human cell lines (HAP)

| Protein | Main location | | | Additional location |
| --- | --- | --- | --- | --- |
| ZDHHC1 | cytosol (approved) | |  | |
| ZDHHC2 | plasma membrane (approved) |  | | |
| ZDHHC3 | Golgi apparatus (approved) | |  | |
| ZDHHC4 | NA' | | | NA' |
| ZDHHC5 | plasma membrane (approved) | | | nucleoplasm (approved) |
| ZDHHC6 | NA' | | | NA' |
| ZDHHC7 | Golgi apparatus (supported) | |  | |
| ZDHHC8 | cytosol (approved) | | | nucleoplasm (approved) |
| ZDHHC9 | ER (supported); Golgi apparatus (supported) | | | cytosol (approved) |
| ZDHHC11 | Mitochondria (approved) | |  | |
| ZDHHC12 | nucleoplasm (approved) | | | Intermediate filaments (uncertain) |
| ZDHHC13 | Vesicles (supported) | | | Golgi apparatus (approved) |
| ZDHHC14 | Nucleoli (approved) | | | Mitochondria (approved) |
| ZDHHC15 | Nuclear speckles (approved) | | | cytosol (approved) |
| ZDHHC16 | nucleoplasm (supported) | | | Nuclear membrane (approved), cytosol (approved) |
| ZDHHC17 | Golgi apparatus (supported); Vesicles (supported) | |  | |
| ZDHHC18 | microtubules (approved) | |  | |
| ZDHHC19 | NA' | | | NA' |
| ZDHHC20 | Vesicles (approved); plasma membrane (supported) | |  | |
| ZDHHC21 | Golgi apparatus (approved); cytosol (approved) |  | | |
| ZDHHC22 | plasma membrane (enhanced) |  | | |
| ZDHHC23 | nucleoplasm (approved) |  | | |
| ZDHHC24 | Vesicles (approved); cytosol (approved) |  | | |

HPA, the Human Protein Atlas (HPA) database; NA', there was no data.

# Supplementary Figures

**
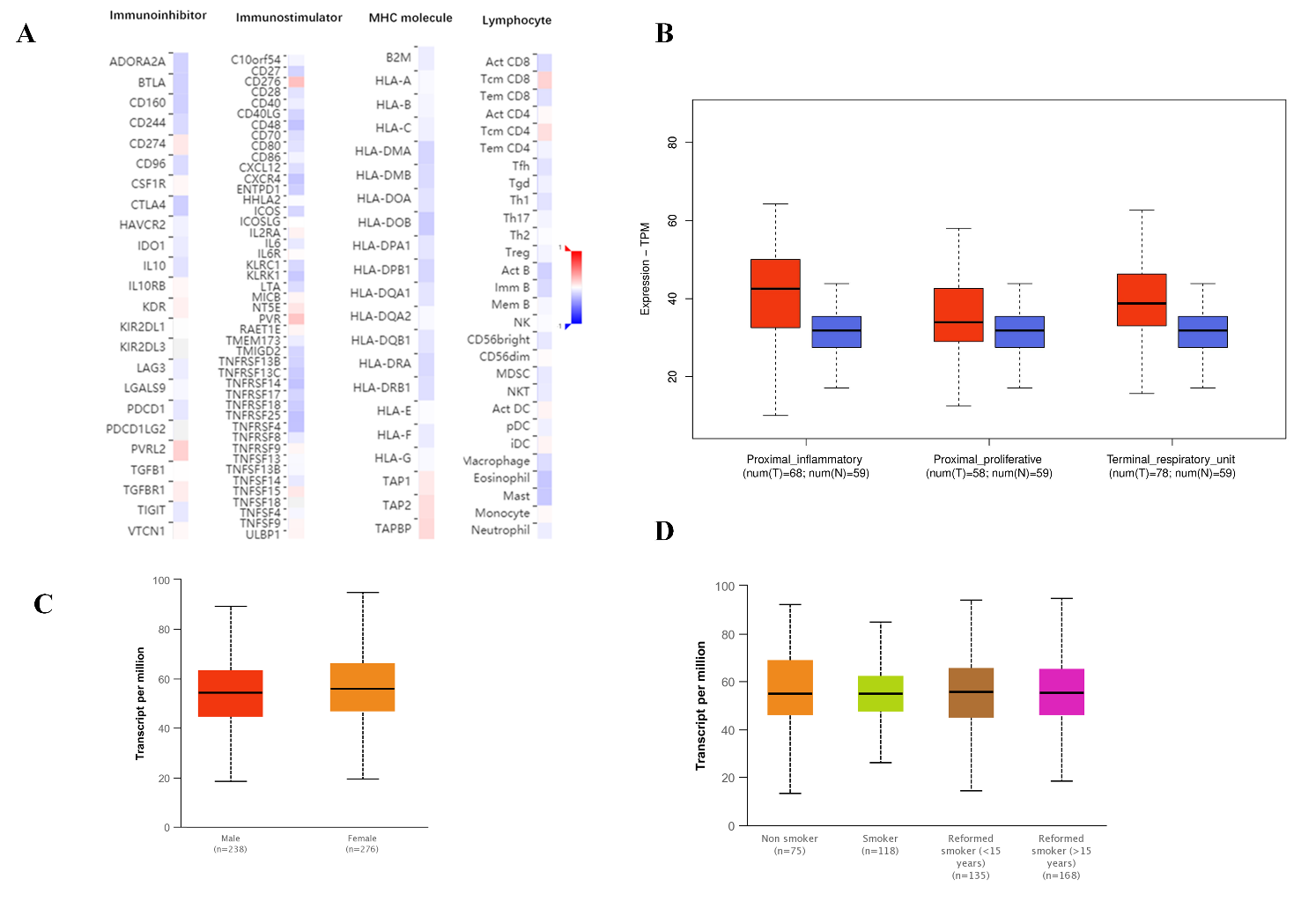
Supplementary Figure 1.** **(A)** Heat map showing the Spearman correlation of ZDHHC5 mRNA expression and immunomodulators and lymphocytes (TISIDB). The color of scale bar presents positive (red) and negative (blue) association respectively, and the intensity of color indicated the value of Spearman coefficient. **(B)** Box plot shows there is no significantly differential expression of ZDHHC5 in LUAD molecular subtypes (*P* > 0.05). **(C)** Box plot showing the gene expression of ZDHHC5 in different genders (*P* > 0.05). **(D)** Box plot showing the differential gene expression of ZDHHC5 in LUAD groups of different smoking habits (*P* > 0.05).

**
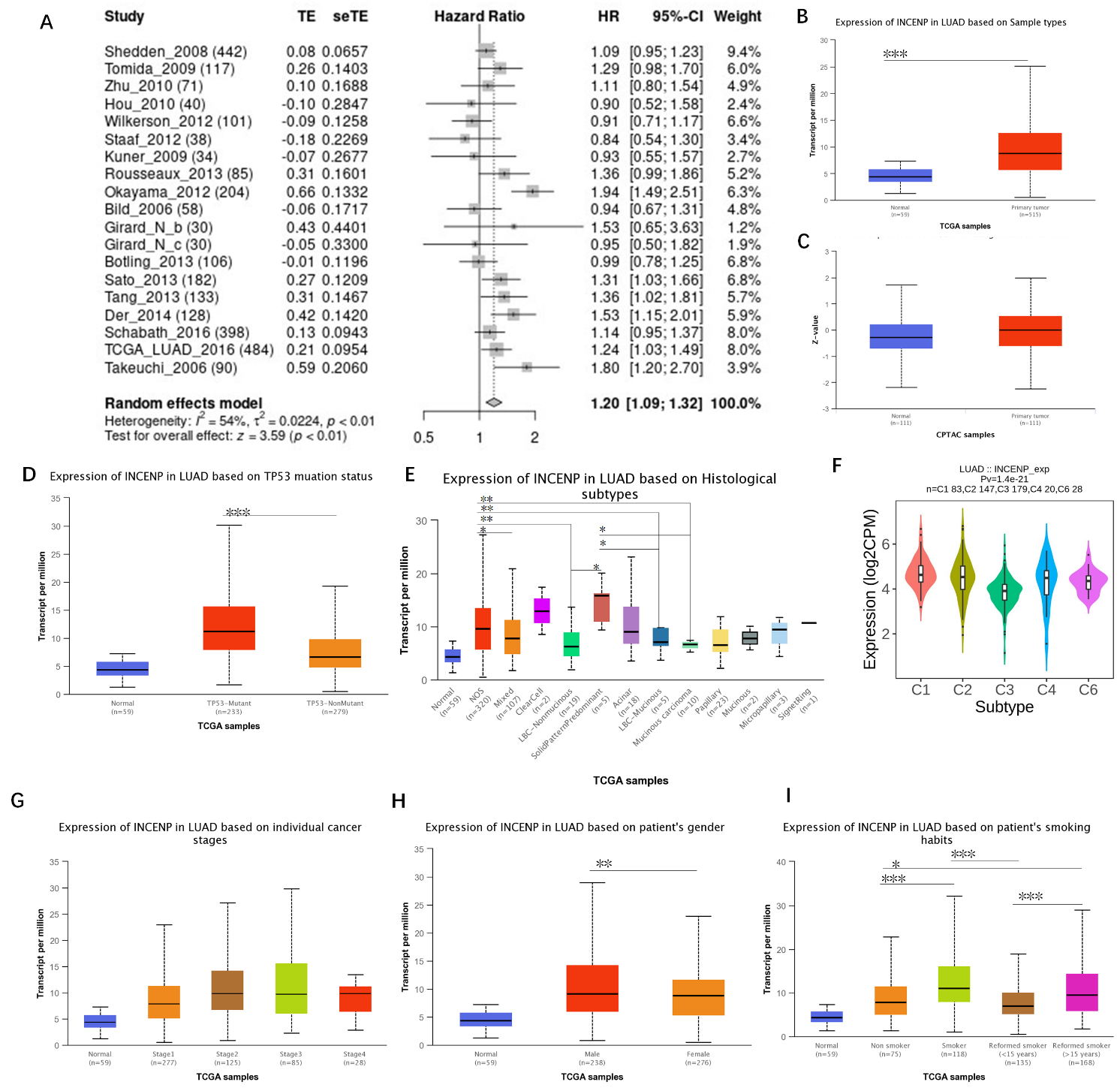
**

**Supplementary Figure 2.** INCENP expression and prognostic value in LUAD. (A) The forest plot indicates the meta-analysis results of INCENP survival probability in 19 individual LUAD studies. The summarized hazard ratio (HR) = 1.2, p < 0.01. (B, C) Differential gene expression of INCENP in LUAD. All data is obtained from TCGA (B) and CPTAC (C) datasets. Box plots show the up-regulation of INCENP in LUADs at the level of both mRNA and protein. The over expression of INCENP mRNA is statistically significant, however the protein expression is not. (D) Box plot showing significant over-expression of INCENP in TP53 mutation LUAD cases compared to the nonmutant group. (E) Box plot showing the relative transcription of INCENP in histological subtypes of LUAD patients. (F) The mRNA expression of INCENP in LUAD cases across immune subtypes. There is a decline in the C3 (inflammatory) subtype. *p < 0.05, **p < 0.01, ***p < 0.001.

**
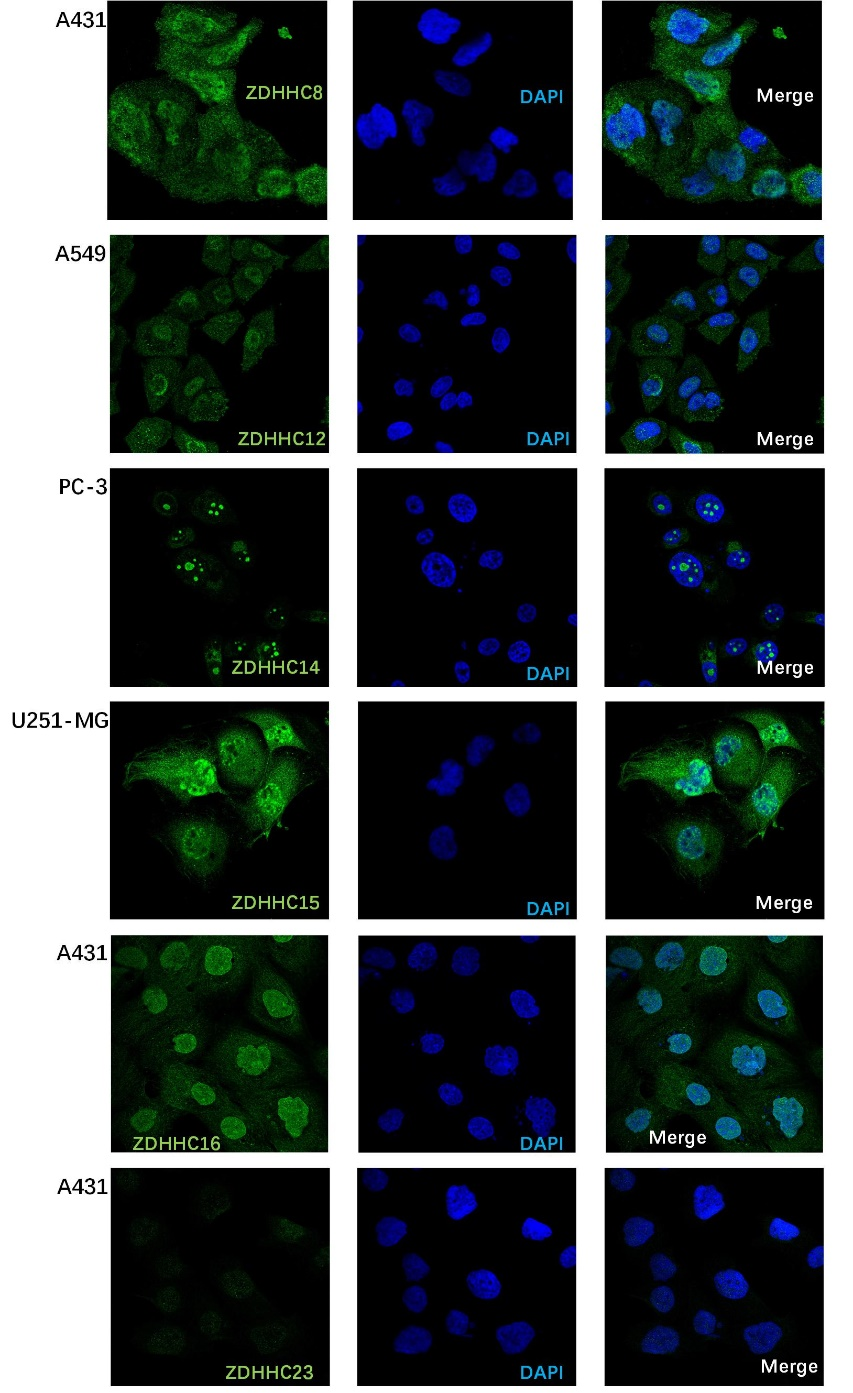
**

**Supplementary Figure 3.** Confocal images show the annotation of the subcellular localization of 6 nuclear ZDHHC proteins (green). The cells are also stained with reference the markers of DAPI (blue) for the nucleus. ZDHHC8 mainly localized to the cytosol, in addition localized to the nucleoplasm. ZDHHC12 mainly localized to the nucleoplasm and the intermediate filaments. ZDHHC14 mainly localized to the nucleoli and the mitochondria. ZDHHC15 mainly localized to the nuclear speckies and the cytosol. ZDHHC16 mainly localized to the nucleoplasm and in addition to localized to the nuclear membrane and cytosol. ZDHHC23 is localized to the nucleoplasm. A549, lung adenocarcinoma cell line; A431, epidermoid carcinoma cell line; PC-3, metastatic poorly differentiated prostate adenocarcinoma cell line; U-251 MG: Glioblastoma cell line.

**Material and methods**

**Immune analysis**

Relations between three kinds of immunomodulators (Immunoinhibitor, Immunostimulator and MHC molecule) were calculated in TISIDB. These immunomodulators were collected from Charoentong's study [1]. Relations between abundance of tumor-infiltrating lymphocytes (TILs) and gene expression performed. The immune-related signatures of 28 TIL types from Charoentong's study. The relative abundance of TILs was inferred by using gene set variation analysis (GSVA) based on gene expression profile.

**Reference**

1. Charoentong P, Finotello F, Angelova M, Mayer C, Efremova M, Rieder D *et al*. Pan-cancer Immunogenomic Analyses Reveal Genotype-Immunophenotype Relationships and Predictors of Response to Checkpoint Blockade. Cell Rep. 2017; 18(1): 248-262.
